# Accuracy Assessment of Using Rapid Prototyping Drill Templates for Atlantoaxial Screw Placement: A Cadaver Study

**DOI:** 10.1155/2016/5075879

**Published:** 2016-11-28

**Authors:** Shuai Guo, Teng Lu, Qiaolong Hu, Baohui Yang, Xijing He, Haopeng Li

**Affiliations:** ^1^Department of Orthopaedics, Second Affiliated Hospital of Xi'an Jiaotong University, Xi'an, Shaanxi Province 710004, China; ^2^Department of Orthopaedics, Second Hospital of Yulin City, Yulin, Shaanxi Province 719000, China

## Abstract

*Purpose.* To preliminarily evaluate the feasibility and accuracy of using rapid prototyping drill templates (RPDTs) for C1 lateral mass screw (C1-LMS) and C2 pedicle screw (C2-PS) placement.* Methods.* 23 formalin-fixed craniocervical cadaver specimens were randomly divided into two groups. In the conventional method group, intraoperative fluoroscopy was used to assist the screw placement. In the RPDT navigation group, specific RPDTs were constructed for each specimen and were used intraoperatively for screw placement navigation. The screw position, the operating time, and the fluoroscopy time for each screw placement were compared between the 2 groups.* Results.* Compared with the conventional method, the RPDT technique significantly increased the placement accuracy of the C2-PS (*p* < 0.05). In the axial plane, using RPDTs also significantly increased C1-LMS placement accuracy (*p* < 0.05). In the sagittal plane, although using RPDTs had a very high accuracy rate (100%) in C1-LMS placement, it was not statistically significant compared with the conventional method (*p* > 0.05). Moreover, the RPDT technique significantly decreased the operating and fluoroscopy times.* Conclusion.* Using RPDTs significantly increases the accuracy of C1-LMS and C2-PS placement while decreasing the screw placement time and the radiation exposure. Due to these advantages, this approach is worth promoting for use in the Harms technique.

## 1. Introduction

In 2001, Harms and Melcher first reported [1] the application of a rod-screw system (RSS) technique in posterior atlantoaxial fixation (AAF) and many other RSS techniques have since been developed [2]. Due to the excellent rigid internal fixation and fewer intraoperative complications of RSS techniques compared with other posterior AAF techniques, these strategies have become increasingly popular in AAF surgeries over the past decade [3–5]. However, the potential for neurovascular injury caused by a malpositioned screw remains a major challenge for surgeons. A recent meta-analysis [6] regarding the screw-related complications of RSSs shows that the screw malposition rate of the C1 lateral mass screw (C1-LMS, Harms technique) [1] is 2.5%. Some malpositioned screws can breach the wall of the lateral mass and encroach into the transverse foramen and spinal canal if the screw is placed too laterally or medially [7, 8]. In addition, when the C1-LMS entry point is too low, the possibility of C2 nerve root dysfunction significantly increases [5, 7, 9]. The malposition rate is also poor for C2-PS fixation. Bransford et al. [10] reported that 18.5% of PSs perforated the cortical bone when lateral intraoperative X-ray and anatomical landmarks were used for guiding screw placement (conventional guided method). Elliott et al. [11] conducted a meta-analysis regarding the malpositioning of C2 screws and found that the incidence of malpositioned PS-related vertebral artery injury (VAI) was 1.09%. Thus, it is imperative to improve the screw placement accuracy in RSS fixation to prevent severe postoperative complications.

Over the past 2 decades, rapid prototyping drill templates (RPDTs) have been used for assisting screw placement in AAF surgeries [12–20]. By using computed tomography (CT) scanning data, reverse engineering software programmes, and rapid prototyping techniques, surgeons can make patient-specific RPDTs and use them for identifying the optimal entry point and angle during the screw placement procedure. As RPDT structures improve, RPDTs continue to significantly increase the accuracy of screw placement in atlantoaxial and atloidooccipital fixation, including Magerl screw, laminar screw, and PS placement [14–20]. However, regarding the application of RPDTs in C1 screw placement, there have been only 2 studies testing the accuracy of using RPDTs for placing screws via posterior arch (VLMS) navigation [21, 22]. Furthermore, we [23] have not found any specific studies assessing the application of the RPDT technique for C1-LMS placement. There are also few studies assessing the accuracy of using RPDTs for C2-PS placement navigation [16, 19, 21, 22]. Thus, in the present study, we aim to first evaluate the feasibility and accuracy of using RPDTs for C1-LMS placement navigation and further evaluate the accuracy of using RPDTs for C2-PS placement navigation.

## 2. Materials and Methods

### 2.1. Specimens

In all, 23 formalin-fixed craniocervical cadaver specimens (average donor age: 65.1 ± 15.3 years; 16 males and 7 females) were obtained with permission from the Department of Anatomy and Pathology of Xi'an Jiaotong University. The 23 cadaver specimens were randomly divided into two groups: the conventional method group (*n* = 11) and the RPDT navigation group (*n* = 12). Thin-layer CT scans (0.625 mm) and 3D reconstruction images of all the craniocervical cadaver specimens were obtained preoperatively to observe the atlantoaxial anatomy of each specimen. In the RPDT navigation group, the CT scans of each craniocervical cadaver specimen were further exported as digital imaging and communications in medicine (DICOM) files, which were used for constructing the RPDTs.

### 2.2. RPDT Construction

**Figure 1:**
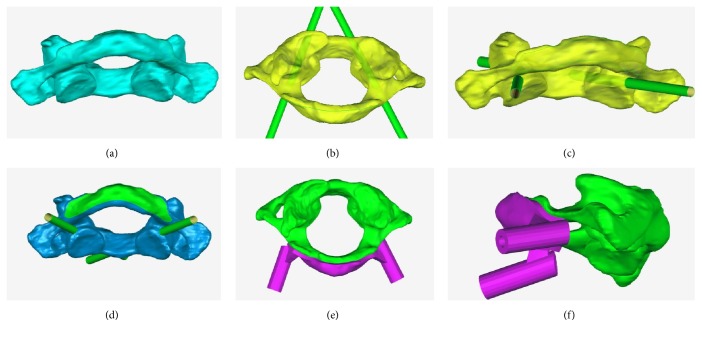
Construction workflow of a C1-LMS RPDT.

**Figure 2:**
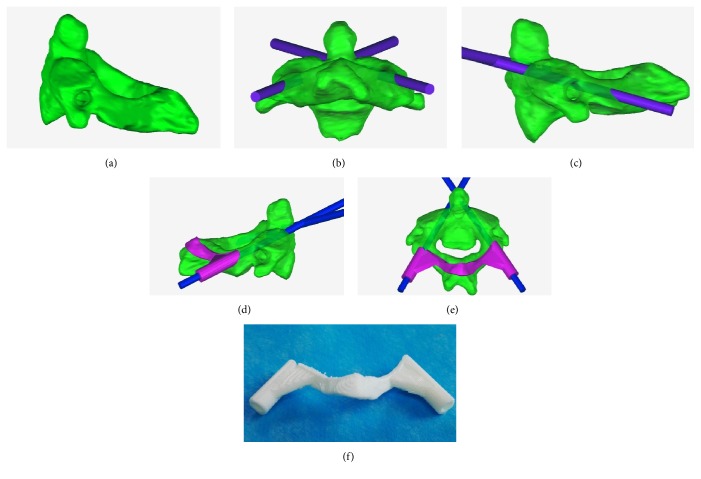
Construction workflow of a C2-PS RPDT.

The DICOM images of each specimen were imported into Mimics 15 (Materialise, Belgium). The image thresholds were set to 226–3071 to mark the bony structures. By using the “Edit Masks,” “Region Growing,” and “Calculate 3D” tools to edit the images, high-quality 3D models of the C1 and C2 were obtained (Figures 1(a) and 2(a)). In addition, a cylinder 4 mm in diameter was constructed with the “MedCAD” tool to simulate the screws.

The 3D models of the C1, C2, and cylinder were exported as stereolithography files and then imported into Geomagic Studio 12 (Geomagic, USA). The “Advanced Object Mover” tool was used to design the optimal position of the cylinder, simulating the optimal screw entry point and angle (Figures 1(b)-1(c) and 2(b)-2(c)). Then, after the partial bony surface of the targeted spinal segment was extracted (Figure 1(d)), the “Shell” tool was used to thicken the extracted bony surface and the partial cylinder (Figures 1(e)-1(f) and 2(d)-2(e)). Subsequently, 3D RPDT models were constructed, and the physical RPDT models were created using a fused deposition modelling (FDM) 3D printer (Waston, China) (Figure 2(f)).

### 2.3. Screw Placement Technique

**Figure 3:**
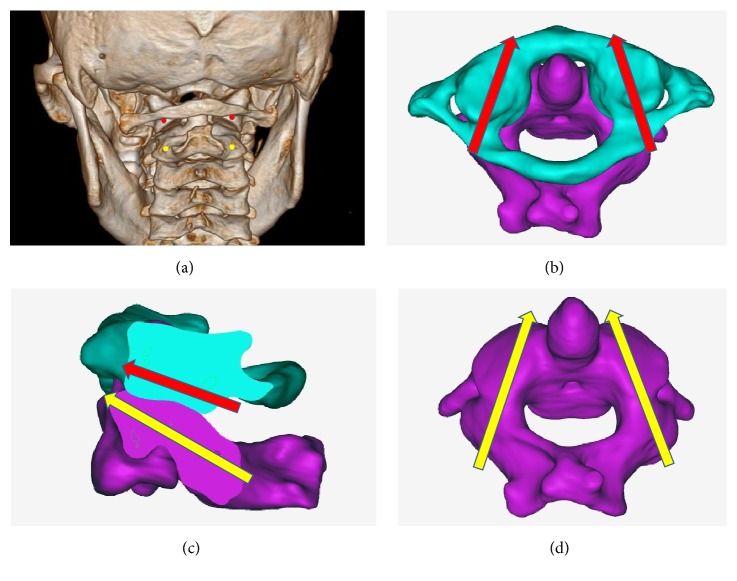
The C1-LMS and C2-PS entry points and directions. (a) The C1-LMS entry point was in the middle of the junction of the C1 posterior arch and the midpoint of the posterior inferior part of the C1 lateral mass (red). The C2-PS entry point was the midpoint between the superior and inferior articular processes (yellow). (b) The C1-LMS was placed in a slightly medial trajectory in the axial plane (red arrows). (c) The C1-LMS was placed parallel to the plane of the posterior arch of the C1 in the sagittal plane (red arrow), and the C2-PS was placed 15–30° cephalad (yellow arrow). (d) The C2-PS was placed 20–25° medially in the axial plane.

**Figure 4:**
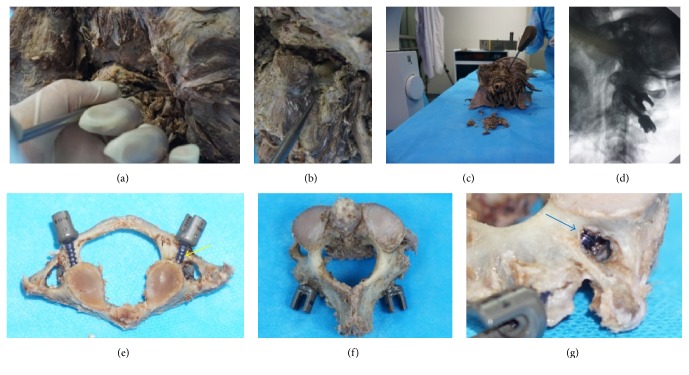
Screw placement using the conventional method and the postoperative assessment of the screw positions.

In the conventional method group, after the posterior atlantoaxial bony structures of the cadaver specimens were exposed, the surgeons (Li and He) used Harms technique [1] to place the C1-LMSs and PSs into the C1 and C2 (Figure 3). During the screw placement procedure, lateral intraoperative X-rays were used to assist in identifying the correct screw entry point and angle (Figures 4(c)-4(d)). The operating and fluoroscopy times for each screw placement were recorded. The operating time began to be recorded when the surgeons began to drill the screw hole, and the operating time ended when the screw insertion was finished. The fluoroscopy times were also recorded for each entry point identification, screw hole direction adjustment, and screw position examination.

**Figure 5:**
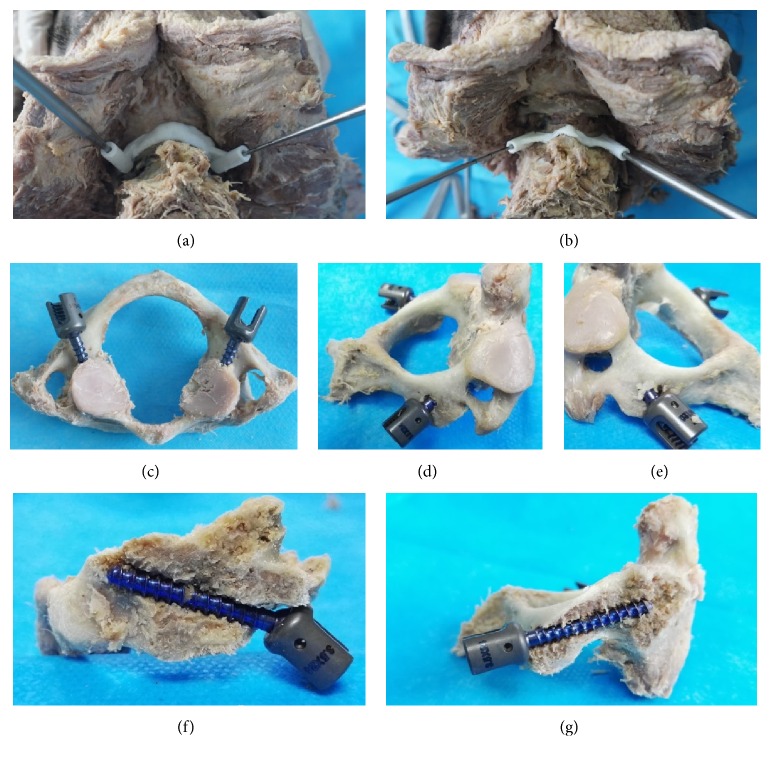
Screw placement using RPDT navigation and the postoperative assessment of the screw positions.

In the RPDT navigation group, aside from exposing the posterior atlantoaxial bony structures of the specimen as in the conventional method group, the soft tissue on the posterior part of the C1 and the lamina and spinous process of the C2 was removed to completely expose the bony surfaces. Then, the RPDT was placed on the corresponding bony surface. At this point, the surgeons ensured that the RPDTs fit properly and stably on the bony structures, that is, made lock-and-key contact. The screw holes were drilled via RPDT navigation (Figures 5(a)-5(b)), and then the screws were inserted into the targeted vertebrae. During the screw placement procedure, lateral intraoperative X-rays were used to evaluate the screw positions only after all the screws had been insertions. The operating and fluoroscopy times for each screw placement were recorded. The recorded operating time began when the surgeons began to place the RPDT on the bony surface, and the time ended when the screw insertion was finished. In each cadaver cervical specimen, as fluoroscopy was used only after all 4 screws were placed, the recorded fluoroscopy times included the examination of the screw positions.

### 2.4. Postoperative Assessment of Screw Placement

After all the screws were inserted into the targeted vertebrae, the C1 and C2 were separated from the specimen, and the authors directly observed and assessed the positions of the screws.

**Figure 6:**
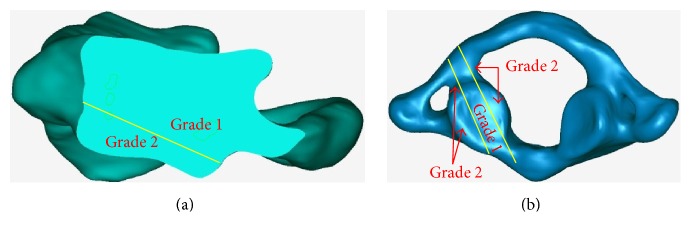
The classifications of C1-LMSs in the sagittal (a) and axial (b) planes.

The positions of the C1-LMSs were assessed in the axial and sagittal plane. In the axial plane, the positions of the C1-LMSs were classified into the following 3 grades (Figure 6(b)):

- Grade 1: the entry point of the C1-LMS is projected onto the middle 50% of the junction of the C1 posterior arch, and the trajectory of the C1-LMS is in the middle 50% of the corresponding lateral mass
- Grade 2: the entry point of the C1-LMS is projected onto the peripheral 50% of the junction of the C1 posterior arch, and the trajectory of the C1-LMS is in the peripheral 50% of the corresponding lateral mass
- Grade 3: the entry point of the C1-LMS is projected onto the external portion of the junction of the C1 posterior arch, or the screw perforates the cortical bone of the C1 lateral mass

In the sagittal plane, the positions of C1-LMSs were also classified into 3 grades, as follows (Figure 6(a)):

- Grade 1: the entry point and trajectory of the C1-LMS are in the upper 50% of the posterior inferior portion of the C1 lateral mass
- Grade 2: the entry point and trajectory of the C1-LMS are in the lower 50% of the posterior inferior portion of the C1 lateral mass
- Grade 3: the entry point of the C1-LMS is under the inferior articular process of the C1 lateral mass, or the screw perforates the cortical bone of the C1 lateral mass

The positions of the C2-PSs were classified into the following 4 grades [16, 24]:

- Grade 1: the screw is completely within the pedicle and the centrum
- Grade 2: the screw perforates the cortical bone of the vertebra, but more than 50% of the screw diameter remains within the vertebra
- Grade 3: the screw perforates the cortical bone of the vertebra and more than 50% of the screw diameter is outside the vertebra
- Grade 4: the screw completely perforates the cortical bone of the vertebra

### 2.5. Statistical Analysis

The data are presented as the mean ± SD, and SPSS 18.0 was used for the statistical analysis. Independent-sample *t*-tests were used to analyse differences in the operating and fluoroscopy times of screw placement between the conventional method group and the RPDT navigation group. The Mann–Whitney *U* test was used to analyse the differences in screw placement accuracy between the 2 groups. *p* value of less than 0.05 was considered statistically significant.

## 3. Results

In the conventional method group, a total of 22 C1-LMSs and 22 C2-PSs were placed into the targeted vertebras. In the axial plane, 16 C1-LMSs (72.7%) were classified as grade 1, and 4 (18.1%) were classified as grade 2. Two C1-LMS (9.2%) were classified as grade 3 because the entry point was too lateral. In the sagittal plane, 20 C1-LMSs (90.1%) were classified as grade 1, and 2 (9.9%) were classified as grade 2. No C1-LMSs were classified as grade 3. Regarding C2-PS placement, 16 C2-PSs (72.7%) were completely inside the cortical bones and were classified as grade 1. Four (18.3%) and 1 (4.5%) C2-PSs partially perforated the cortical bones and were classified as grades 2 and 3, respectively. One C2-PS (4.5%) was completely inside the transverse foramen and was classified as grade 4 (Table 1).

In the RPDT navigation group, a total of 24 C1-LMSs and 24 C2-PSs were placed into the targeted vertebrae. In the axial plane, all the C1-LMSs (100%) were in the middle of the lateral mass and classified as grade 1. In addition, in the sagittal plane, all the C1-LMSs (100%) were in the upper 50% of the posterior inferior portion of the C1 lateral mass and classified as grade 1. No C1-LMSs were classified as grade 2 or 3. Regarding the C2-PS placement, 23 C2-PSs (95.8) were completely inside the cortical bones and classified as grade 1. There was 1 C2-PS (95.8%) that partially perforated the cortical bones and was classified as grade 2. The analysis of the outcomes showed that the application of RPDTs improved the accuracy of C2-PS and C1-LMS placement in the axial plane compared with the conventional method. However, RPDTs did not significantly increase the accuracy of C1-LMS placement in the sagittal plane (Table 1).

The average operating time for screw placement in the RPDT navigation group was 2.3 ± 0.76 minutes (range: 1 to 3.5 minutes), which was significantly shorter than that in the conventional method group (mean: 4.5 ± 1.39 minutes; range: 2.5 to 6 minutes). The average fluoroscopy time for each screw placement in the RPDT navigation group was 0.4 ± 0.8 (range: 1 to 3 for each cadaver specimen), which was also significantly lower than that in the conventional method group (mean: 3.1 ± 1.01, range: 1 to 5 for each screw placement) (Table 2).

## 4. Illustrative Cases

### 4.1. Case  1

AAF surgery was performed using the conventional method in a 61-year-old female craniocervical cadaver specimen (Figure 4). After the bony structures of the craniocervical region were exposed, the surgeons inserted the C1-LMSs and C2-PSs with the assistance of lateral intraoperative X-rays (Figure 4(e)). After all the screws were inserted, the C1 and C2 were separated from the craniocervical cadaver to assess the screw positions. The postoperative outcomes showed that the entry point and trajectory of 1 C1-LMS were projected onto the peripheral 50% of the junction of C1 posterior arch and the lateral mass, which was classified as grade 2 in the axial plane (Figures 4(a)–4(d)). One C2-PS completely perforated the pedicle and severely penetrated the transverse foramen, which was classified as grade 4 (Figures 4(f)-4(g)).

### 4.2. Case  2

AAF surgery was performed in a 54-year-old male craniocervical cadaver specimen using RPDTs for screw placement navigation (Figure 5). After the soft tissue on the posterior part of the C1 and the lamina and spinous process of the C2 was completely removed, the surgeons placed the RPDTs on the corresponding bony surfaces and drilled the entry points and trajectories under the navigation of the RPDTs (Figures 5(a)-5(b)). After all the screws were inserted, the C1 and C2 were separated from the craniocervical cadaver specimen to assess the screw positions. The postoperative outcomes showed that the positions of all the screws were optimal and were classified as grade 1 (Figures 5(c)–5(g)).

## 5. Discussion

In Harms technique, malpositioned screws can occasionally cause severe complications [6]. Gunnarsson et al. [7] used postoperative CT scanning to observe the position of C1-LMSs and found that 1 C1-LMS severely encroached into the spinal canal when it was placed too medially. Yeom et al. [8] also reported that vertebral artery occlusion resulted from 1 screw breaching the medial wall of the lateral mass by 5 mm and 1 screw encroaching into the left transverse foramen. In addition, malpositioned screws may often cause C2 nerve root dysfunction, and patients may suffer from C2 nerve numbness or neuropathic pain postoperatively due to a C1-LMS entry point that was too low [5, 7, 9]. Related studies have also reported high C2-PS malposition rates [8, 10]. Some of these malpositioned screws have caused severe neurovascular complications [11]. Thus, it is very important to improve screw placement accuracy and avoid screw-related complications in AAF surgeries.

Over the past 2 decades, RPDTs have been applied in AAF surgeries to help surgeons place screws accurately [12–20]. Goffin et al. [12, 13] first applied the RPDT technique to Magerl screw placement; however, the screw placement accuracy outcomes were disappointing. These results were mainly because the RPDT only had small points of contact with the bony structures, which resulted in the RPDT not being stable enough to cope with the drilling forces, thereby altering the designed entry point and trajectory [12, 13]. Subsequently, surgeons began to design a new type of RPDT that had sufficient contact with the corresponding lamina and spinous process to obtain enough stability to resist the drilling forces during the screw placement procedures. This new type of RPDT led to a significantly increased screw placement accuracy. Kawaguchi et al. [17] first used this type of RPDT for Magerl screw placement navigation. The postoperative CT images of their case series showed that 95.4% of the Magerl screws were completely within the cortex of the bone and that no screw-related neurovascular injuries occurred. Other studies [14, 16] that evaluated the feasibility of this RPDT in Magerl screw placement also showed a very high accuracy (100%), which demonstrated the applicability of the surface-contact template structure.

Other screw fixation techniques in AAF surgeries also benefit from the RPDT technique, including VPAS [21, 22], PS [16, 19, 21], and laminar screw fixation [15, 16, 20]. Lu et al. [19] first tested the accuracy and reliability of RPDTs in C2-PS fixation. Among the enrolled patients, 4 patients had very narrow pedicles, with a minimum diameter of 3.5 mm. The postoperative CT images of all the enrolled patients showed that none of the screws perforated the cortex of the bone, which preliminarily demonstrated the high accuracy and reliability of RPDTs. Kaneyama et al. [16] also obtained a high accuracy rate (97.9%) of C2-PS placement using RPDTs for navigation. They [16] concluded that RPDTs facilitated precise screw insertion and significantly simplified the placement procedure, especially in patients with craniovertebral deformities and narrow pedicles, whose screw entry point and variable vertebrae angle are difficult to identify using conventional methods. Hu et al. [21] first used an RPDT to perform VPAS fixation in cervical cadaver specimens. Postoperative CT images of all the 64 VPASs showed that none of the screws breached the cortical bone. In addition, there were no significant differences in the deviations of the entry point location or screw orientation between the designed and actual trajectories. Sugawara et al. [22] first used an RPDT for VPAS navigation in clinical practice. Postoperative CT scans also showed that all the screws were in optimal positions and did not penetrate the cortical bone. Additionally, no screw-related complications occurred during the operations, which demonstrated the high accuracy and safety of using RPDTs for screw placement.

A well-designed RPDT structure is critical for outstanding navigation accuracy. Aside from the surface-contact structure, some other design tips should be included in the RPDT construction workflow. First, when performing the preoperative CT scan of the target vertebrae, thin-layer CT scanning must be used because it yields more detailed information about the bony surface, which is essential for constructing a highly accurate 3D model of the target vertebrae [21]. Second, an RPDT that is fitted to a single vertebra is recommended; it eliminates the influence of any movement among the adjacent vertebrae [25]. Third, compared with the unilateral structure, a bilateral structure is more appropriate because the surface area in contact with the vertebra increases, rendering it more stable during the screw hole drilling procedure [14]. Based on these design features, a high-quality RPDT can be constructed. In the present study, the C1 RPDT was designed to only have contact with the posterior arch. Considering that the position of the lateral mass is relatively deep and that the venous plexus and C2 nerve root are in this region, a space between the lateral mass and the drill guide was included in the design to adequately expose the operative field and avoid neurovascular injuries during the screw placement procedure.

In the present study, 2 C1-LMSs were placed too laterally in the axial plane in the conventional method group, which could carry a potential risk for VAI. In contrast, using the RPDT technique for navigation, all of the C1-LMSs were placed in the middle of the C1 lateral mass, indicating that RPDTs enable highly accurate navigation and decrease the risk of neurovascular injuries in C1-LMS placement. In the sagittal plane, there were no significant differences between the RPDT navigation and conventional method groups. However, all the screws in the RPDT group were optimally positioned, that is, in the upper 50% of the posterior inferior portion of the C1 lateral mass, which also demonstrated the applicability of the RPDT technique for protecting the C2 nerve root from screw-related injuries [5, 7, 9]. One common complication during C1-LMS placement is venous plexus bleeding [5, 7]. However, as this study was conducted using cadaver specimens, whether the use of an RPDT has an impact on the incidence of venous plexus bleeding is difficult to assess. Thus, the feasibility of applying the RPDT technique in C1-LMS placement in clinical practice should be further assessed.

Regarding the position assessment of the C2-PSs, 23 of 24 screws (95.8%) in the RPDT group were completely inside the pedicle and the cortical bone of the centrum, which was a significantly greater proportion than that in the conventional method group (72.7%). In the RPDT group, 1 screw deviated from the designed trajectory and partially perforated the corresponding pedicle (grade 2). In this case, we rechecked the vertebra and found some soft tissue on the corresponding C2 lamina, which caused the RPDT to fit the C2 improperly, providing an incorrect navigation direction to the surgeons. Thus, it is imperative to completely remove the soft tissue on the corresponding bony surfaces to ensure that the RPDT properly fits the vertebra and provides an accurate navigation direction to the surgeons. In addition to absolutely remove the soft tissue to ensure optimal RPDT accuracy, some other precautions should also be noted to obtain optimal navigation results. One is that the RPDT should be constructed to be more than 2 mm in thickness; otherwise the RPDT may be too soft and deform during the screw hole drilling procedure, which may result in an incorrect screw hole direction being drilled. The other is that, during the screw hole drilling procedure, the RPDT should be fixed on the bony surface by hands or forceps such that it cannot move and change the navigational direction.

Studies evaluating the accuracy of RPDTs in AAF surgeries have used CT scans as the gold standard for assessing screw position because CT scans can clearly demonstrate the spatial relationship between the screws and the vertebrae [12–22]. However, in the present study, we applied a novel evaluation method, which used vertebral dissection for grading this relationship. By using this method, the internal structure of the vertebrae and the screw position can be clearly observed, and surgeons can observe the spatial relationship between the screw and the vertebrae at every viewing angle, which is sufficiently accurate and objective to grade the screw position. Additionally, we used this method because the cost of CT scans for all 23 craniocervical cadaver specimens was quite expensive, and the cost was greatly decreased by using vertebral dissection for this assessment.

As shown in the results, in addition to providing a high navigation accuracy, the RPDT technique also significantly decreased the operating time and radiation exposure compared to the conventional method. When using the conventional method for screw placement, surgeons usually need to use intraoperative X-rays to repeatedly evaluate the position of the hand drill or the screw, repeatedly adjusting the entry point and trajectory to obtain the best position; this process increases the radiation exposure of the patients and surgeons as well as the length of the operation [26]. By using an RPDT for navigation, the procedure for identifying the trajectory is greatly simplified because the RPDT already indicates the optimal entry point and angle for the surgeons to drill the screw hole.

The RPDT technique also has some limitations. One limitation is that constructing an RPDT is time consuming [12, 15, 19, 21, 27]. In this study, 2 or 3 days were required to complete the construction workflow, from collecting the cervical CT data to obtaining a 3D physical model of the RPDT. Another disadvantage is that surgeons need to invest a great amount of time to become proficient with the software, which can be burdensome. These limitations may be key factors restricting the widespread use of the RPDT technique. Thus, it is imperative to simplify the RPDT construction workflow to further promote this technique.

## 6. Conclusion

The use of an RPDT significantly increases the accuracy of C1-LMS and C2-PS placement. Meanwhile, this technique can also decrease both screw placement time and radiation exposure. Due to these advantages, the RPDT approach is worth promoting for use in Harms technique.

## Figures and Tables

**Table 1:** Comparison of screw placement accuracy between the conventional method and RPDT navigation groups (Mann–Whitney *U* test).

Grade	C1-LMS (axial plane)		C1-LMS (sagittal plane)		C2-PS	
	Conventional method	RPDT navigation	Conventional method	RPDT navigation	Conventional method	RPDT navigation
1	16 (72.7%)	23 (95.8%)	20 (90.1%)	24 (100%)	16 (72.7%)	23 (95.8%)
2	4 (18.1%)	1 (4.2%)	2 (9.9%)	0 (0%)	4 (18.3%)	1 (4.2%)
3	2 (9.2%)	0 (0%)	0 (0%)	0 (0%)	1 (4.5%)	0 (0%)
4	—	—	—	—	1 (4.5%)	0 (0%)
Total	22	24	22	24	22	24
*Z* value	2.185		1.494		2.185	
*p* value	0.029		0.135		0.029	

**Table 2:** Comparison of operating and fluoroscopy times between the conventional method and RPDT navigation groups (independent-sample *t*-tests).

	Operating time (min)		Fluoroscopy times	
	Conventional method	RPDT navigation	Conventional method	RPDT navigation
Mean ± SD	4.5 ± 1.39	2.3 ± 0.76	3.1 ± 1.01	0.4 ± 0.8
Sample size	44	48	44	48
*t* value	9.751		13.963	
*p* value	<0.001		<0.001	
